# Meta-analysis of clinical risk factors of patients with systemic lupus erythematosus complicated with invasive fungal infection

**DOI:** 10.1097/MD.0000000000029652

**Published:** 2023-03-17

**Authors:** Yang Meng, Liao Chifeng, Zhu Qinghuan, Huang Zichan, Li Jianmin, Deng Danqi

**Affiliations:** a Department of Dermatology, The Third Affiliated Hospital of Guangxi Medical University, Nanning, Guangxi, China; b Department of Dermatology, The Second Affiliated Hospital of Kunming Medical University, Kunming, Yunnan, China; c Institute of Dermatology and Department of Dermatology, No.1 Hospital, Anhui Medical University, Hefei, Anhui, China.

**Keywords:** IFI, meta-analysis, SLE, systematic review

## Abstract

To systematically evaluate the clinical risk factors of patients with systemic lupus erythematosus (SLE) complicated with invasive fungal infection (IFI) among patients.

**Methods:**

A meta-analysis was performed of all the literatures germane to estimate the clinical risk factors of patients with SLE complicated with IFI from published clinical trials from 1990 to April 2022. Mean differences, odds ratio and 95% confidence intervals were calculated, and the meta-analysis was conducted with Stata 12.0 software (StataCorp, College Station, TX).

**Results:**

A total of 14 clinical research involving 1129 patients were included. The results of meta-analysis demonstrated that immunosuppressant, glucocorticoids, systemic lupus erythematosus disease activity index score, antibiotic were risk factors associated with IFI in SLE patients. However, age, sex, course of disease, leukopenia, lymphopenia, C- reactive protein and hypoproteinemia were not the risk factors associated with IFI in patients with SLE.

**Conclusion:**

Our results indicate that immunosuppressant, glucocorticoids, systemic lupus erythematosus disease activity index score, antibiotic were risk factors for IFI in SLE patients. However, high quality of multicenter, large sample size-controlled trials are needed to validate the result.

## 1. Introduction

Systemic lupus erythematosus (SLE) is a common autoimmune disease which characterized by a loss of self-tolerance, the production of autoantibodies and inflammation of multiple organs. SLE has a wide variety of clinical symptoms, which involve multiple organs: skin, serosa, joints, kidneys, central nervous system, and so on. There are a variety of autoantibodies in the patient, which affects not only humoral immunity, but also cellular immunity, and changes in the complement system. The pathogenesis is mainly due to the formation of immune complexes. The exact cause is unknown. The disease showed an alternating process of recurrence and remission. The disease is more common in young women. In Global, the prevalence of SLE in adults range from 30 to 150 per 100,000, and incidence ranges from 2.2 to 23.1 per 100,000 per year.^[1]^ Currently, glucocorticoids (GCs) and/or immunosuppressant are widely used in the treatment of SLE. Those treatment can improve the clinical response rate and survival time. However, due to the widespread use of GCs and immunosuppressants and immune disorders caused by the disease itself, the secondary infection is one of the main complications and main causes of death in patients with SLE, with a mortality rate of about 30%.^[2]^

Invasive fungal infection (IFI) has become an increasingly serious problem, with the prevalence of IFIs varying from 0.83%^[3]^to 4.8% in different populations.^[4]^ It is usually caused by different types of fungi, such as Cryptococcus, Aspergillus, Candida, histoplasmosis.^[5]^ At present, there are a large number of clinical studies on SLE concurrent fungal infection. Most of researches reported that a systemic lupus erythematosus disease activity index (SLEDAI) 2K score higher than 8 points, the increase of titer of anti-DNA antibody, neutropenia, lymphocytopenia, use of steroid and antibiotic, mechanical ventilation and hemodialysis have some correlation with IFI.^[6]^ However, a retrospective study by Weng^[7]^ showed that 50% of SLE patients with IFI did not have a higher SLEDAI score. Moreover, most of the above studies come from relatively independent single-center studies, and the results of the risk factors of the studies are not completely consistent, and the results of domestic and foreign studies are also different. Therefore, the purpose of this study is to systematically analyze the related risk factors of SLE concurrent IFI through relevant research reports at home and abroad, so as to early prediction, diagnosis and intervention for IFI in clinic.

## 2. Methods

We included case-control studies of IFI risk in SLE patients. The outcomes of our meta-analysis were age, sex, course of disease, immunosuppressant, glucocorticoids, antibiotic, leukopenia, lymphopenia, C- reactive protein (CRP), hypoproteinemia, and SLEDAI Score.

### 2.1. Literature search and selection

Searches were applied to the following electronic databases: MEDLINE, EMBASE, and the Cochrane Library, Chinese Biomedical Database, China National Knowledge Infrastructure, WANFANG and Chinese social sciences citation index databases without language restrictions. The search strategy was based on the combinations of “Lupus erythematosus or lupus nephritis or LE” and “fungal infection or IFI or invasive fungal infections or fungus infection.” We also modified the terms according to the different databases. Last query was updated on 15 April 2022. References of retrieved articles were cross-searched to identify any studies missed by the electronic search strategies.

### 2.2. Inclusion criteria

Inclusion and exclusion criteria for original studies were as follows:

Firstly, only clinical trials of IFI group comparing with non-IFI for SLE were included regardless of follow up, language or publication status. Secondly, the articles must be proven diagnosis of SLE patients with standard criteria of ACR criteriain in 1997. There were also clear diagnostic criteria for fungal infection. Diagnosis of fungal infections: there are signs and symptoms of fungal infections, which have the cause of infection and meet at least one of the following 3 items: Positive blood fungal culture; puncture bone marrow or closed chamber to take liquid microscopy for fungi or fungal culture Positive, or positive antigen reaction; Sputum, urine, stool and local secretions fungus culture more than 2 times to detect the same fungus. Moreover, when multiple articles were published by the same authors or institutions, the most recent or informative single article was selected. Thirdly, articles lacking original data for meta-analysis, review articles were excluded.

### 2.3. Data extraction

Our initial selection of all candidate articles was relied on careful screening of their abstracts by 2 independent reviewers, using a standardized data collection form, including the following items: the first author, year of publication, sex, mean or median age, course of disease, immunosuppressant, glucocorticoids, antibiotic, leukopenia, lymphopenia, CRP, hypoproteinemia, and SLEDAI Score and assessment of outcomes.

We manually searched the reference lists of the found articles. We also screened references from the relevant literature, including all of the identified studies, but no additional reviews and editorials. Disagreements were resolved by consensus between the 2 readers. In case of persistent disagreement, the final decision was made by our expert.

The quality of the nonrandomized studies was assessed by using the Newcastle-Ottawa Scale with some modifications to match the needs of this study.^[8]^ The quality of the studies was evaluated by examining 3 items: patient selection, comparability of groups, and assessment of outcome. Studies were graded on an ordinal star scoring scale with higher scores representing studies of higher quality. A study can be awarded a maximum of 1 star for each numbered item within the selection and exposure categories and a maximum of 4 stars can be given for the comparability of the 2 groups. The quality of each study was graded as either level 1 (0 to 5) or level 2 (6 to 9).

### 2.4. Statistical methods

All statistical analyses were performed using Statistical Analysis System software (Stata 12.0; StataCorp, College Station, TX), and the *P* value for the overall effect < .05 with 2-tailed was considered statistically significant. The heterogeneity of all involved studies was assessed by *I*^2^. When it was lower than 50%, the studies with an acceptable heterogeneity were considered, and then the fixed-effects model with Mantel-Haenszel method was used, otherwise, a random effect model with the Der Simonian and Laird method was adopted. The combined odds ratio (OR) and Mean difference (MD) were initially estimated using Forrest plots. For each trial, the OR and MD was estimated from the original article. If it is not available, we looked at the total numbers of events and the numbers of patients at risk in each group to determine the OR and MD estimate.

Assessment of publication bias was investigated for each of the pooled study groups mainly by the Egger linear regression test. As supplement approach, the Begg rank correlation also was applied to assess the potential publication bias, when *P* < .05 was considered that there was no publication bias in the study.

## 3. Results

### 3.1. Literature search and eligible studies

**Figure 1.**
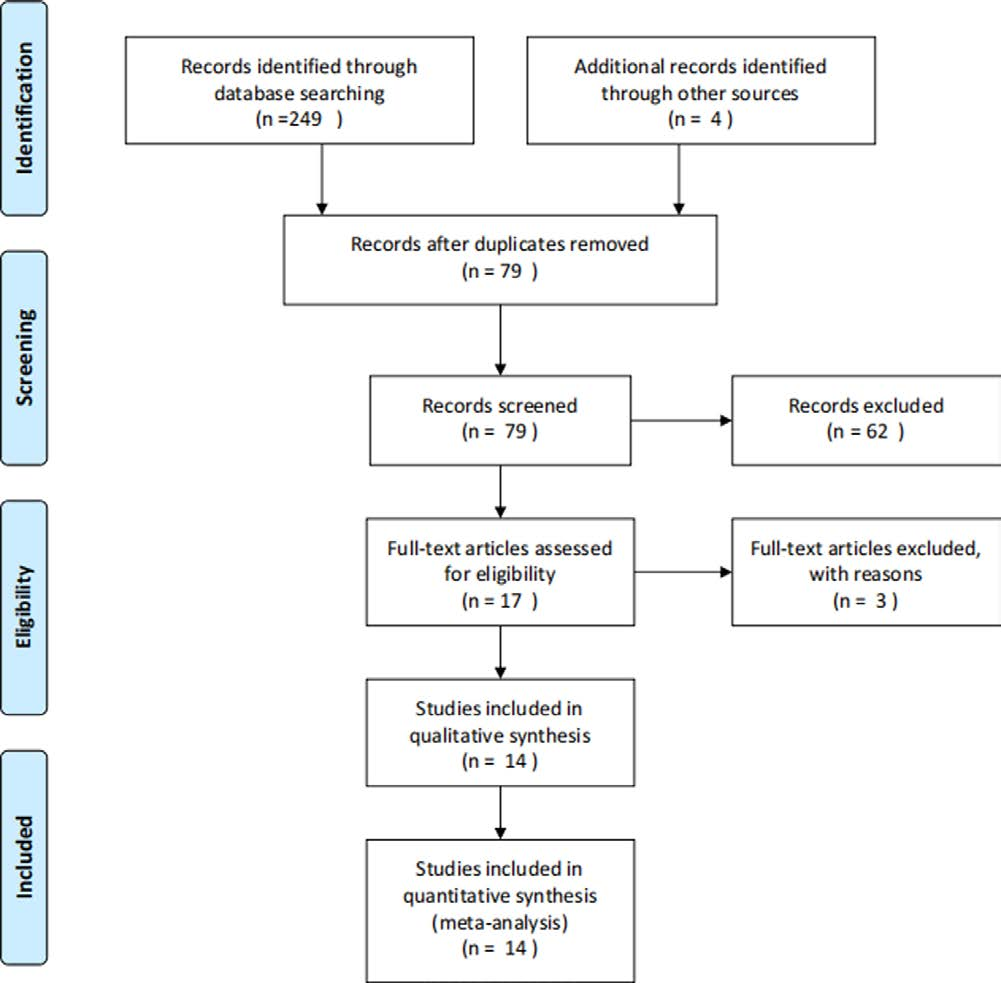
Selection process for the studies included in the meta-analysis.

A total of 253 references were retrieved for initial review using search strategies as described. Seventy-nine citations were excluded from analysis after the first screening based on abstracts or titles. After exclusion of the articles that were out of the scope of our meta-analysis, we identified 17 potential studies for detail evaluation. Upon further review, 2 studies had no control group, and the diagnostic criteria of 1 study was unclear. Finally, 14 studies were performed on the IFI group versus non-IFI in patients with SLE. Selection process for the studies included in the meta-analysis were summarized in Figure 1. The main features of eligible studies in our meta-analysis were summarized in Table 1.

**Table 1:** Main characteristics of the 14 studies included in the final meta-analysis.

Included studies	Published time	Region	M/F (R)	M/F (C)	Average age (R/C, yr)	Course of research, yr	Follow up time	Outcomes	NOS score
Yang CD^[9]^	2007	Shanghai, China	NS	NS	34.3 ± 11.1/30.1 ± 11.1	10 yr	NS	①②③④⑤⑩	7
Liu XY^[20]^	2011	Guangzhou, China	NS (19)	NS (101)	NS	5 yr	NS	④⑤⑥	7
MARCO ULISES^[10]^	2012	San Luis Potosí, México	3/7	9/41	30.5/28.5	7 yr	NS	①②③④⑥⑦⑧⑨⑪	9
Chen GL^[15]^	2012	Shanghai, China	2/16	8/82	30.8 ± 11.6/32.4 ± 13.7	4 yr	NS	②④⑤⑥⑪	8
Deng HX^[11]^	2013	Hunan, China	5/43	4/37	34.8 ± 11.2/32.6 ± 10.5	4 yr	NS	①②③⑨⑩⑪	7
JP Vinicki^[5]^	2013	Buenos Aires, Argentina	2/8	6/24	27.5/28	11 yr	NS	①②③④⑤⑥⑦⑧⑪	9
Rao L^[16]^	2013	Hebei, China	12/60	100/514	58.08 ± 9.11/NS	3 yr	NS	②④⑩	8
Fan R^[12]^	2014	Shandong, China	4/18	6/30	45 ± 19/37 ± 22	3 yr	NS	①②④⑥⑦⑨⑪	8
Marco F^[17]^	2015	São Paulo, Brazil	4/29	116/703	14.5/17	2 yr	NS	②⑨⑪	
Tan Z^[18]^	2015	Anhui, China	18/129	14/133	37.09 ± 11.31/36.32 ± 9.17	9 yr	NS	②③④⑤⑥	8
Tan X^[21]^	2016	Hunan, China	NS (27)	NS (27)	36.74 ± 17.01/39.59 ± 12.41	4 yr	NS	⑤⑩	8
Guo Y^[19]^	2017	Yunnan, China	NS	NS	NS	4 yr	NS	②④⑤⑥⑩	7
Santamaría-Alza^[13]^	2018	Bucaramanga, Colombia	4/11	27/158	34.66 ± 15.84/38.09 ± 14.86	5 yr	NS	①②③④⑤⑨⑪	9
Lao M^[14]^	2019	Guangdong, China	10/35	13/77	37.2 ± 14.3/33.1 ± 12.6	10 yr	NS	①②⑦⑧⑨⑩⑪	9

‘M’ and ‘F’ stands for male and female respectively; “R” and “C” corresponds to research group and control group respectively; “NS” stands for not stated; ①age; ②sex; ③course of disease; ④ Immunosuppressant; ⑤Glucocorticoids; ⑥ Antibiotic; ⑦ Leukopenia; ⑧ Lymphopenia; ⑨C- reactive protein (CRP); ⑩ Hypoproteinemia; ⑪SLEDAI.

NOS = Newcastle-Ottawa scale.

### 3.2. Meta-analysis results

#### 3.2.1. Age.

**Figure 2.**
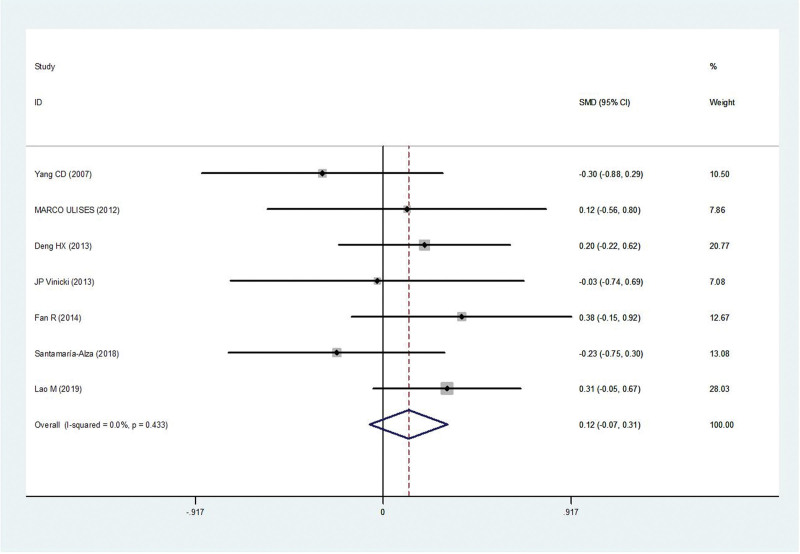
Meta-analysis of age about SLE with IFI compared with those without IFI. IFI = invasive fungal infection, SLE = systemic lupus erythematosus.

Seven studies^[5,9–14]^ reported age in patients with IFI compared with those without IFI (163 patients and 512 controls). There was no statistical heterogeneity among the studies (*P* = .43, *I*^2^ = 0%). Thus, the fixed-effect model was used for statistical analysis. Age was not the risk factors associated with IFI in patients with SLE (MD = 0.12, 95% confidence intervals [CI] −0.07, 0.31, *P* = .20) (Fig. 2).

#### 3.2.2. Sex.

**Figure 3.**
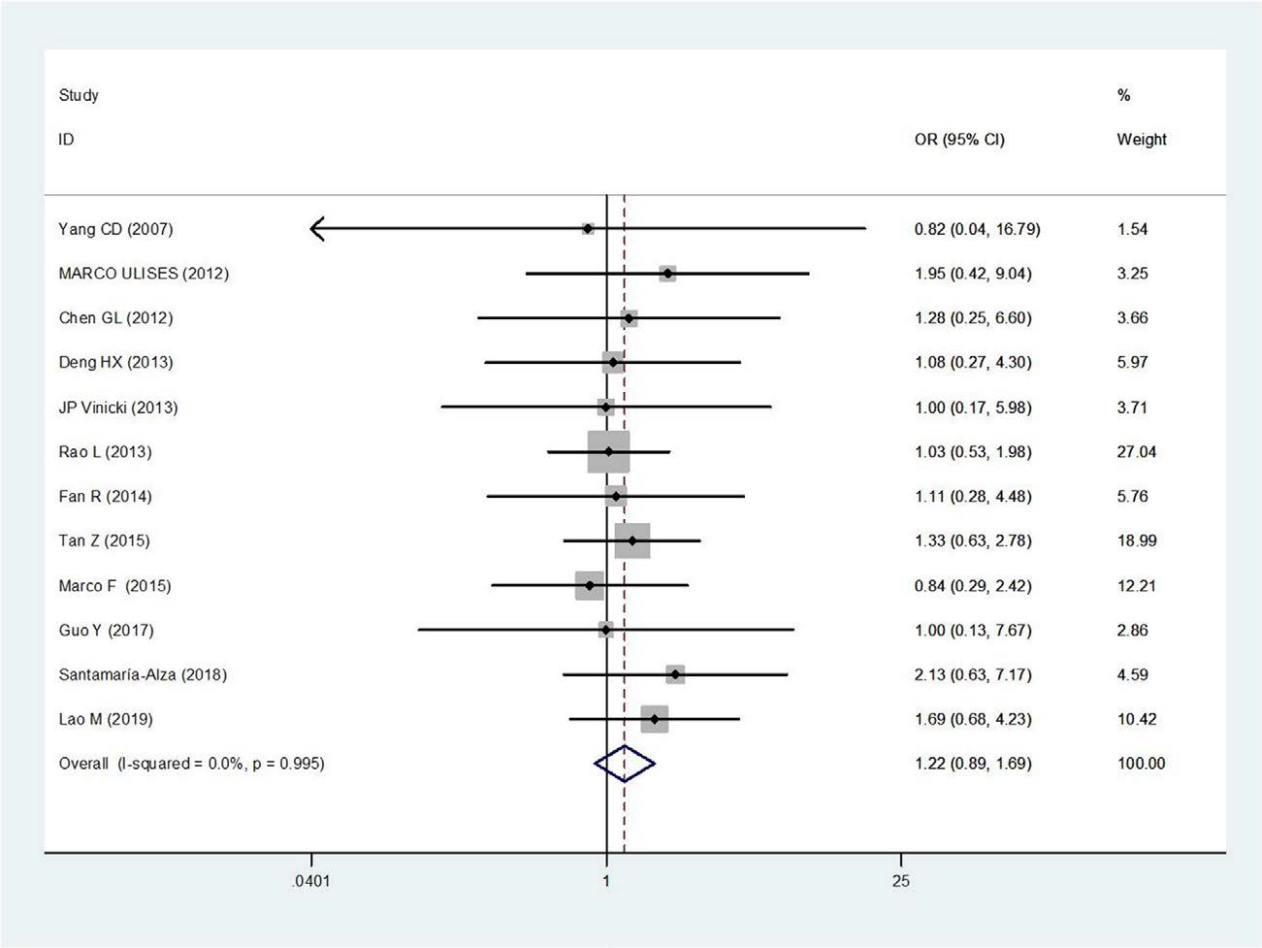
Meta-analysis of sex about SLE with IFI compared with those without IFI. IFI = invasive fungal infection, SLE = systemic lupus erythematosus.

Twelve studies^[5,9–19]^ reported sex in patients with IFI compared with controls (250 males and 1901 females). There was no statistical heterogeneity among the studies (*P* = .99, *I*^2^ = 0%). Thus, the fixed-effect model was used for statistical analysis. Sex was not the risk factors associated with IFI in SLE patients (OR = 1.22, 95% CI 0.89, 1.69, *P* = .22) (Fig. 3).

#### 3.2.3. Course of disease.

Six studies^[5,9–11,13,18]^ reported course of disease in patients with IFI compared with controls. There was no statistical heterogeneity among the studies (*P* = .30, *I*^2^ = 18.3%). Thus, the fixed-effect model was used for statistical analysis. Course of disease was not the risk factors associated with IFI in SLE patients (OR = 0.03, 95% CI −0.14, 0.20, *P* = .73).

#### 3.2.4. Immunosuppressant.

**Figure 4.**
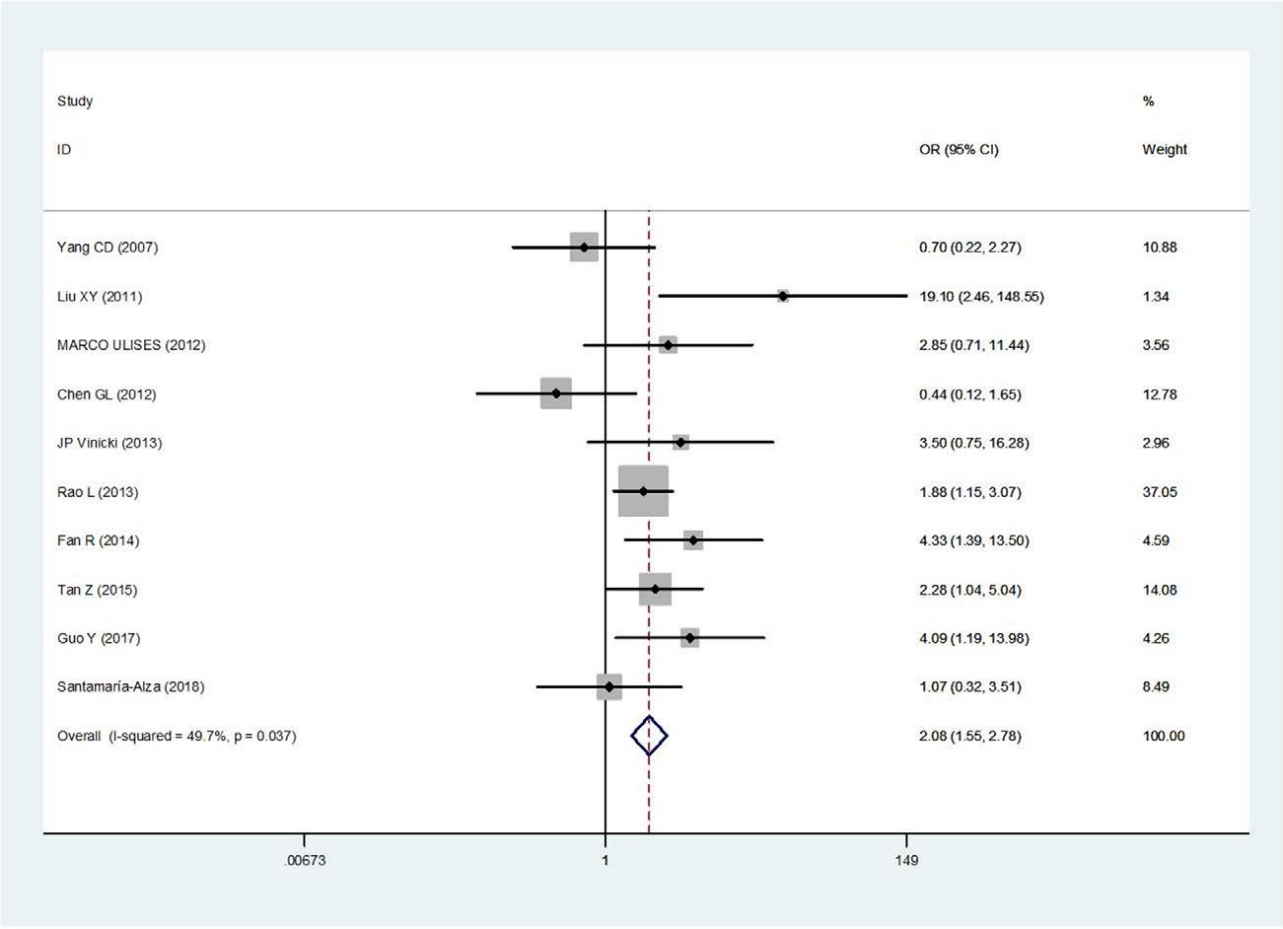
Meta-analysis of immunosuppressant about SLE with IFI compared with those without IFI. IFI = invasive fungal infection, SLE = systemic lupus erythematosus.

The immunosuppressant was reported in ten studies (SLE + IFI vs SLE alone).^[5,9,10,12,13,15,16,18–20]^ There was no obvious statistical heterogeneity among the studies (*P* = .04, *I*^2^ = 49.7%). Thus, the fixed-effect model was used for statistical analysis. Immunosuppressant was the risk factors associated with IFI in SLE patients (OR = 2.08, 95% CI 1.55, 2.78, *P* < .001) (Fig. 4).

#### 3.2.5. GCs.

**Figure 5.**
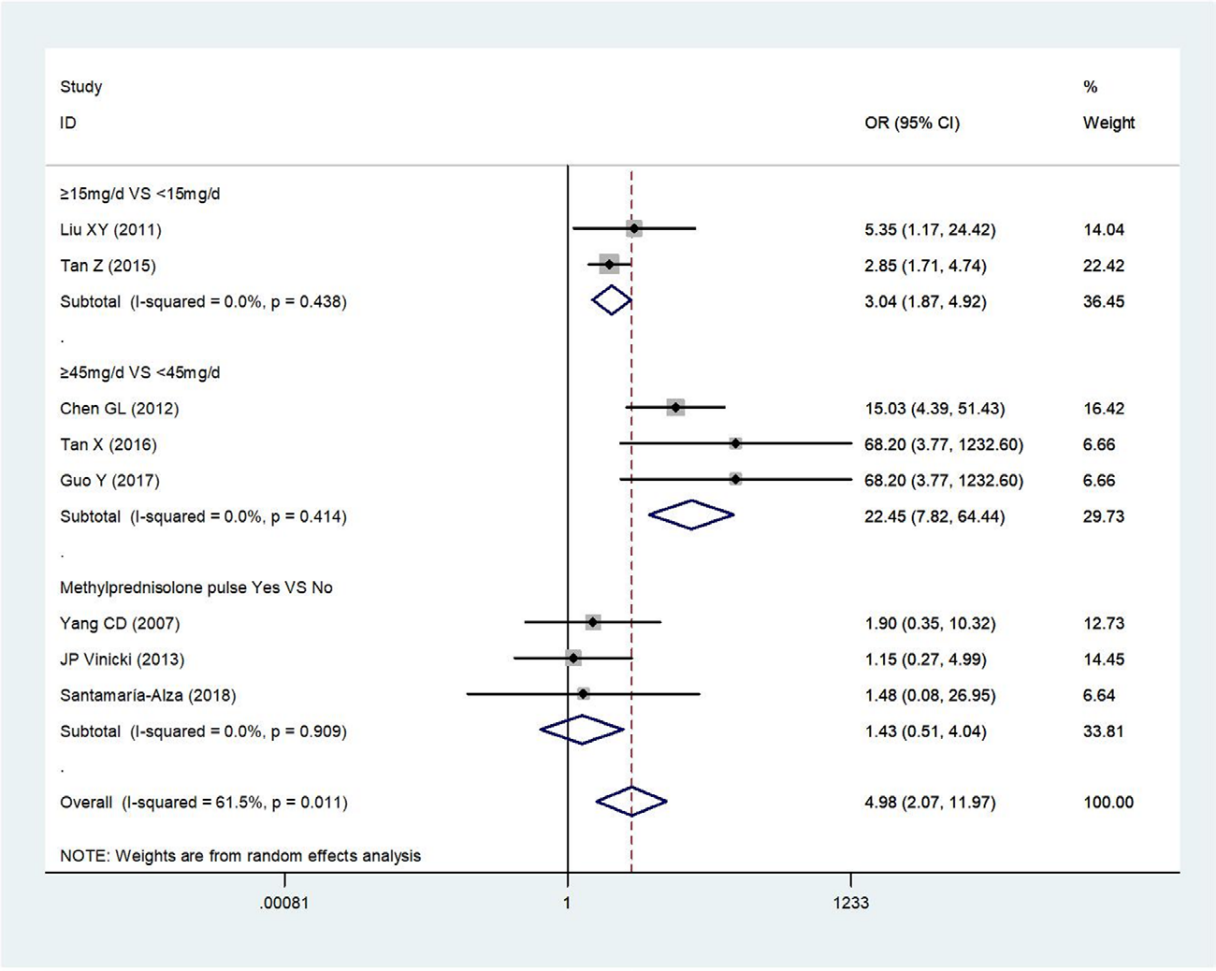
Meta-analysis of glucocorticoids about SLE with IFI compared with those without IFI. IFI = invasive fungal infection, SLE = systemic lupus erythematosus.

Eight studies^[5,9,13,15,18–21]^ reported GCs in patients with IFI compared with controls. There was statistical heterogeneity among the studies (*P* = .01, *I*^2^ = 61.5%). Thus, the random model was used for statistical analysis. And we divided it into 3 subgroups according to the dose of GCs. GCs dosage >15 mg/day was risk factor associated with IFI in patients with SLE (≥15mg/day vs < 15 mg/day) (OR = 3.04, 95% CI 1.87, 4.92, *P* < .001).And GCs dosage >45mg/day was the obvious risk factor associated with IFI in patients with SLE (≥45 mg/d vs < 45 mg/day) (OR = 22.45, 95% CI 7.82, 64.44, *P* < .001).However, methylprednisolone was not the risk factors associated with IFI in SLE patients (OR = 1.43, 95% CI 0.51, 4.04, *P* = .73) (Fig. 5).

#### 3.2.6. Antibiotic.

**Figure 6.**
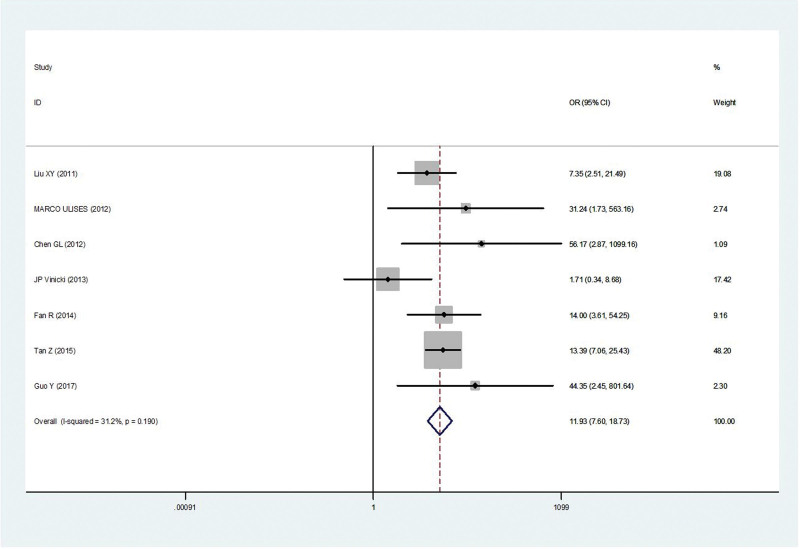
Meta-analysis of antibiotic about SLE with IFI compared with those without IFI. IFI = invasive fungal infection, SLE = systemic lupus erythematosus.

Seven studies^[5,10,12,15,18–20]^ reported antibiotic in patients with IFI compared with controls. There was no statistical heterogeneity among the studies(*P* = .19, *I*^2^ = 31.2%). Thus, the fixed-effect model was used for statistical analysis. Antibiotic was the risk factors associated with IFI in SLE patients (OR = 11.93, 95% CI 7.60, 18.73, *P* < .001) (Fig. 6).

#### 3.2.7. Leukopenia.

Four studies^[5,10,12,14]^ reported leukopenia in patients with IFI compared with controls. There was statistical heterogeneity among the studies (*P* < .05, *I*^2^ = 95.0%). Thus, the random model was used for statistical analysis. Leukopenia was not the risk factors associated with IFI in SLE patients (MD = 0.86, 95% CI −0.52, 2.25, *P* = .22).

#### 3.2.8. Lymphopenia.

Three studies^[5,10,14]^ reported lymphopenia in patients with IFI compared with controls. There was no statistical heterogeneity among the studies (*P* = .15, *I*^2^ = 47.6%). Thus, the fixed-effect model was used for statistical analysis. Lymphopenia was not the risk factors associated with IFI in SLE patients (MD = 0.12, 95% CI −0.17, 0.41, *P* = .41).

#### 3.2.9. CRP.

CRP was reported in 6 studies.^[10–14,17]^ There was statistical heterogeneity among the studies (*P* < .05, *I*^2^ = 98.7%). Thus, the random model was used for statistical analysis. There was no difference in the level of CRP (MD = 0.33, 95% CI −1.61, 2.27, *P* = .74) between the 2 groups.

#### 3.2.10. Hypoproteinemia.

Six studies^[9,11,14,16,19,21]^ reported hypoproteinemia in patients with IFI compared with controls. There was statistical heterogeneity among the studies (*P* < .05, *I*^2^ = 89.9%). Thus, the random model was used for statistical analysis. Hypoproteinemia was not the risk factors associated with IFI in SLE patients (OR = 3.47, 95% CI 0.66, 18.34, *P* = .14).

#### 3.2.11. SLEDAI score.

Eight studies^[5,10–15,17]^ reported the result of SLEDAI Score, there was statistical heterogeneity among the studies (*P* < .05, *I*^2^ = 87.6%). Thus, the random effect model was used for statistical analysis.

**Figure 7.**
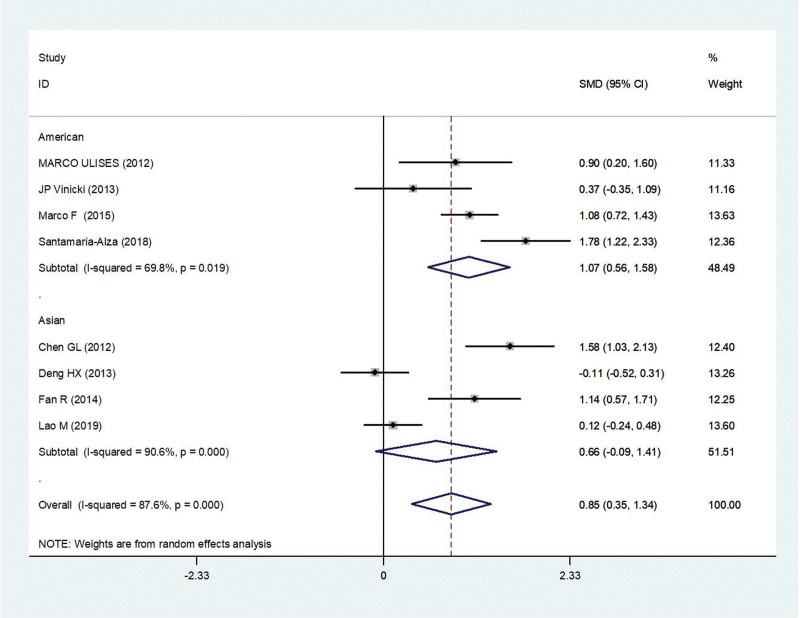
Meta-analysis of SLEDAI Score about SLE with IFI compared with those without IFI. IFI = invasive fungal infection, SLE = systemic lupus erythematosus, SLEDAI = systemic lupus erythematosus disease activity index.

And we divided it into 2 subgroups according to the race. SLEDAI score in American was risk factor associated with IFI in patients with SLE (OR = 1.07, 95% CI 0.56, 1.58, *P* < .001). However, SLEDAI score in Asian was not risk factor associated with IFI in patients with SLE (OR = 0.66, 95% CI −0.09, 1.41, *P* = .09) (Fig. 7). Summary results for the 2 groups were summarized in Table 2.

**Table 2:** Summary of outcomes about SLE with IFI compared with those without IFI.

Outcomes	Subgroup analysis	Number of studies	MD or OR (95% CI)	*P* value	Heterogeneity test	Effect model
Age	No	7	0.12, (−0.07, 0.31)	*P* = .20	*P* = .43, *I*^2^ = 0%	Fixed
Sex	No	13	1.22, (0.89, 1.69)	*P* = .22	*P* = .99, *I*^2^ = 0%	Fixed
Course of disease	No	6	0.03, (−0.14, 0.20)	*P* = .73	*P* = .30, *I*^2^ = 18.3%	Fixed
Immunosuppressant	No	10	2.08, (1.55, 2.78)	*P* < .001	*P* = .04, *I*^2^ = 49.7%	Fixed
Glucocorticoids		8	4.98, (2.07, 11.97)	*P* < .001	*P* = .01, *I*^2^ = 61.5%	Random
	≥15 mg/d vs < 15 mg/d	2	3.04, (1.87, 4.92)	*P* < .001	*P* = .44, *I*²=0%	Random
	≥45 mg/d vs < 45 mg/d	3	22.45, (7.82, 64.44)	*P* < .001	*P* = .41, *I*²=0%	Random
	Methylprednisolone pulse Yes vs No	3	1.43, (0.51, 4.04)	*P* = .73	*P* = .91, *I*²=0%	Random
Antibiotic	No	7	11.93, (7.60, 18.73)	*P* < .001	*P* = .19, *I*^2^ = 31.2%	Fixed
Leukopenia	No	4	0.86, (−0.52, 2.25)	*P* = .22	*P* < .05, *I*^2^ = 95.0%	Random
Lymphopenia	No	3	0.12, (−0.17, 0.41)	*P* = .41	*P* = .15, *I*^2^ = 47.6%	Fixed
C−reactive protein (CRP)	No	6	0.33, (−1.61, 2.27)	*P* = .74	*P* < .05, *I*^2^ = 98.7%	Random
Hypoproteinemia	No	6	3.47, (0.66, 18.34)	*P* = .14	*P* < .05, *I*^2^ = 89.9%	Random
SLEDAI Score		8	0.85, (0.35, 1.34)	*P* = .001	*P* < .05, *I*^2^ = 87.6%	Random
	American	4	1.07, (0.56,1.58)	*P* < .001	*P* = .20, *I*^2^ = 69.8%	Random
	Asian	4	0.66, (−0.09, 1.41)	*P* = .09	*P* < .05, *I*^2^ = 90.6%	Random

CIs = 95% confidence intervals, CRP = C- reactive protein, IFI = invasive fungal infection, MD = mean difference, OR = odds ratio, SLE = systemic lupus erythematosus, SLEDAI = systemic lupus erythematosus disease activity index.

#### 3.2.12. Fungal infection strain and site.

Summarizing all the fungal infection strains and parts in all the literature, the most common top 5 strains are Candida albicans, Cryptococcus neoformans, Aspergillus, Candida tropicalis, and others. The top 5 infection sites are oral cavity, lung, gastrointestinal tract, skin, and central nervous system.

### 3.3. Sensitivity analysis and publication bias

**Figure 8.**
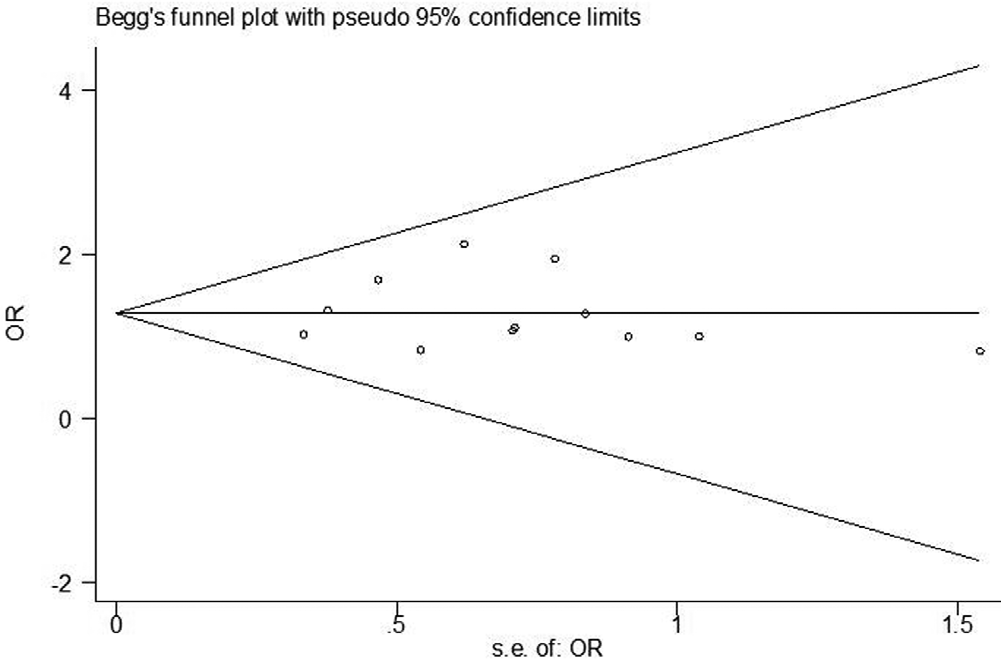
Funnel plot of sex about SLE with IFI compared with those without IFI. IFI = invasive fungal infection, SLE = systemic lupus erythematosus.

The influence analyses revealed that none of the studies significantly affected the pooled MDs, OR or CIs. When each study was sequentially removed and meta-analysis was repeated with the remaining studies, the pooled MD and OR remain nearly the same. Egger linear regression test and Begg test were used to examine publication bias. There was no evidence of publication bias for the analyses of various indicators (e.g., Funnel plot of sex about SLE with IFI compared with those without IFI, Fig. 8).

## 4. Discussion

SLE is a kind of diffuse connective tissue disease with multiple organ involvement, which is mediated by autoimmunity and characterized by immune inflammation. Fungal infections can be seen in people whose immune system is unable to remove the invading pathogens or opportunistic infections caused by the imbalance of flora. Among them, deep fungal infections are mostly secondary to diseases with extremely low immune function or even defects. Due to the damage of this disease and the use of immunosuppressive, the patient is in an immunosuppressive state, which is easy to be complicated by IFI.^[22]^ IFI has the characteristics of insidious onset and atypical clinical manifestations. It is easily covered up by the symptoms and signs of the primary disease, especially when it is complicated by bacterial infections. IFI often occurs early in the course of SLE, accounting for between 0.64% and 3.24% of SLE hospitalized patients.^[23]^ These infections resulted in significant mortality, and many were only diagnosed postmortem.

In this meta-analysis, 11 possible risk factors were analyzed. Part of the study results are consistent with previous clinical analysis, such as age, sex, course of disease, leukopenia, lymphopenia, CRP, and hypoproteinemia are not risk factors for SLE complicated by IFI; SLEDAI score, the use of GCs, immunosuppressant and antibiotics are risk factors for SLE complicated by IFI.

It is worth noting that in this analysis, we divide the GCs into 3 subgroups and found that the use of GCs ≥ 15 mg and ≥ 45 mg were risk factors. The reason may be that GCs can weaken the body’s inflammatory response, affect the redistribution of circulating lymphocytes and lymphocyte function, make the cellular immune function abnormal, reduce the synthesis of immune globulin and complement, and thus affect the body’s defense mechanism. However high-dose pulse of GCs is not a risk factor. The specific reason and mechanism are not yet clear, but in clinical work, we must carefully interpret and use high-dose pulse of GCs, and have good predictability and control of disease treatment, outcome, and prognosis.

The analysis of the SLEDAI score shows the difference between races. SLEDAI is a risk factor in American, but it is not in Asian. This may be related to geographical, ethnic, genetic weight, lifestyle, and other factors.

Studies have shown that patients with overlapping IFI and active SLE are more likely to have leukopenia (40%) or normal leukocyte count (42%) than leukocytosis (17%).^[23]^ And this overlap may lead to a higher SLEDAI score. There are also reports that the leukocytes > 3 × 10^8^/L is risk factors affecting secondary fungal infection in SLE.^[24]^ However, the results of this systematic analysis show that leukopenia is a non-risk factor for SLE complicated with IFI. In addition, lymphopenia, C-reactive protein and hypoproteinemia are also non-risk factors in SLE complicated with IFI. This is inconsistent with the results of some studies. The research by Lao^[25]^ show that lymphopenia (OR = 3.28, 95% CI 1.29–8.38, *P* = .01) was associated with fungal infection in patients with connective tissue disease. This may be related to the location and species of fungi in SLE patients. Therefore, more samples are needed to confirm such inconsistent results.

In addition, we find that immunosuppressant and antibiotics are risk factors for SLE complicated by IFI. Therefore, in the future treatment, it is necessary to pay attention to reducing the use of antibiotics and immunosuppressive agents for SLE patients with potential IFI infection risks.

Due to disease activity, GCs and immunosuppression in SLE patients. The application of fungicides can significantly increase the probability of fungal infection and superficial fungal infection. Oral infection is the most common, while pulmonary infection is the most common. The common pathogen is Candida albicans. SLE combined infections are mainly mixed infections,^[26]^ fungal infections can exist alone, or can be mixed with pathogens such as bacteria and viruses. In the clinical process, according to age, disease course, white blood cell count, neutrophil percentage, 1, 3-β-D- Glucan test (G test), GM (galactomannan) test, full set of virus, purified protein derivative skin test, T-SPOT testing, imaging examination, etiological examination, and other data to identify. In addition to SLE treatment, we need to actively prevent fungal infection. Early diagnosis is needed for suspected cases of infection.

There are some limitations in this study. First, some heterogeneity was observed, but we used subgroup analysis and gave a more reasonable explanation to the results. Second, because of the incorporated literature, including related research at home and abroad, there may be differences in ethnicity, regional culture, and the measurement methods of the same outcome. In addition, some patients with childhood-onset SLE and perimenopausal women were included in this analysis, there may be population selection bias. Third, all included studies were retrospective and observational, with several methodological limitations, and that the pooled studies had different sample sizes and geographic locations. Fourth, for the analysis of hormone risk factors, only 3 subgroups of dose gradient analysis can be performed, and accurate statistical analysis of hormone use time and treatment course cannot be performed. We are looking forward to more high quality of multicenter, large sample size to validate the results.

## 5. Conclusions

Our results indicate that immunosuppressant, GCs, SLEDAI score, antibiotic were risk factors for IFI in SLE patients. However, high quality of multicenter, large sample size-controlled trials are needed to validate the result.

## Author contributions

**Conceptualization:** Danqi Deng.

**Investigation:** Zichan Huang.

**Methodology:** Qinghuan Zhu.

**Software:** Jianmin Li.

**Supervision:** Jianmin Li.

**Validation:** Jianmin Li.

**Visualization:** Danqi Deng.

**Writing – original draft:** Meng Yang.

**Writing – review & editing:** Chifeng Liao.
